# Addressing the gaps in diabetes care in first nations communities with the reorganizing the approach to diabetes through the application of registries (RADAR): the project protocol

**DOI:** 10.1186/s12913-017-2049-y

**Published:** 2017-02-06

**Authors:** Dean T. Eurich, Sumit R. Majumdar, Lisa A. Wozniak, Allison Soprovich, Kari Meneen, Jeffrey A. Johnson, Salim Samanani

**Affiliations:** 1 School of Public Health, University of Alberta, Edmonton, AB T6G 1C9 Canada; 2 Alliance for Canadian Heath Outcomes Research in Diabetes, University of Alberta, Edmonton, AB T6G 2E1 Canada; 3 Department of Medicine, Faculty of Medicine and Dentistry, University of Alberta, Edmonton, AB T6G 2R7 Canada; 4 OKAKI Health Intelligence Inc, #715, 3553 – 31st NW, Calgary, AB T2L 2K7 Canada

**Keywords:** First Nations, Diabetes, Electronic Health Record, Care Coordinator, Registry

## Abstract

**Background:**

Type-2 diabetes rates in First Nations communities are 3-5 times higher than the general Canadian population, resulting in a high burden of disease, complications and comorbidity. Limited community nursing capacity, isolated environments and a lack of electronic health records (EHR)/registries lead to a reactive, disorganized approach to diabetes care for many First Nations people. The Reorganizing the Approach to Diabetes through the Application of Registries (RADAR) project was developed in alignments with federal calls for innovative, culturally relevant, community-specific programs for people with type-2 diabetes developed and delivered in partnership with target communities.

**Methods:**

RADAR applies both an integrated diabetes EHR/registry system (CARE platform) and centralized care coordinator (CC) service that will support local healthcare. The CC will work with local healthcare workers to support patient and community health needs (using the CARE platform) and build capacity in best practices for type-2 diabetes management. A modified stepped wedge controlled trial design will be used to evaluate the model. During the baseline phase, the CC will work with local healthcare workers to identify patients with type-2 diabetes and register them into the CARE platform, but not make any management recommendations. During the intervention phase, the CC will work with local healthcare workers to proactively manage patients with type-2 diabetes, including monitoring and recall of patients, relaying clinical information and coordinating care, facilitated through the shared use of the CARE platform. The RE-AIM framework will provide a comprehensive assessment of the model.

The primary outcome measure will be a 10% improvement in any one of A1c, BP, or cholesterol over the baseline values. Secondary endpoints will address other diabetes care indicators including: the proportion of clinical measures completed in accordance with guidelines (e.g., foot and eye examination, receipt of vaccinations, smoking cessation counseling); the number of patients registered in CARE; and the proportion of patients linked to a health services provider. The cost-effectiveness of RADAR specific to these communities will be assessed. Concurrent qualitative assessments will provide contextual information, such as the quality/usability of the CARE platform and the impact/satisfaction with the model.

**Discussion:**

RADAR combines innovative technology with personalized support to deliver organized diabetes care in remote First Nations communities in Alberta. By improving the ability of First Nations to systematically identify and track diabetes patients and share information seamlessly an overall improvement in the quality of clinical care of First Nations people living with type-2 diabetes on reserve is anticipated.

**Trial registration:**

ISRCTN study ID ISRCTN14359671, retrospectively registered October 7, 2016.

## Background

It is well known that type-2 diabetes disproportionally impacts Indigenous/First Nations people across the world, including Canada [1]. Overall, the prevalence of type-2 diabetes in First Nations communities is 3–5 times higher than in the general Canadian population [2, 3]. First Nations populations experience a high burden of disease, complications, and comorbidity [1], which is compounded by suboptimal care in isolated or remote communities [4–6]. Moreover, First Nations communities consistently rank among the lowest on social determinants of health, eroding their capacity to adopt health-promoting conditions, navigate the health system and advocate for change [7].

**Table 1:** RADAR Structure by the RE-AIM Framework

RE-AIM dimension	Brief definition of dimension	Approach	Data source(s)
Adoption	Willingness of community members to initiate an intervention	Qualitative approach	Document review
Reach	Into the target population(s) (e.g., First Nations members with T2D)	Quantitative approach	Registry/CARE analytics
Implementation	Intervention is implemented as intended, and consistently across communities	Qualitative approaches	Interviews, document review, field notes
Effectiveness	Determined by outcomes	Quantitative approach	Registry/CARE analytics Interviews
Maintenance	Intervention effects or sustainability over time	Qualitative approach	Document review

Integral to the delivery of organized diabetes care are the 5R’s: **R**egister (systematically tracking all patients); **R**elay (facilitate information sharing); **R**ecall (timely review and reassessment); **R**ecognize (screening/risk factors assessment); and **R**esource (support self-management) [8]. In Canada, there is a shift towards a more comprehensive chronic care model (CCM) [8], including in the management of type-2 diabetes. However, most people living in First Nations communities have limited access to regular primary care and specialist services required for CCM-consistent diabetes care [9], with most care delivered through federally funded, nurse-led homecare and community health programs [10]. Perhaps due to fragmented care, along with cultural and geographical barriers, and physician/nursing shortages in rural and remote communities, First Nations populations have poor diabetes-related outcomes, including high rates of dialysis, blindness, amputations and foreshortened life expectancy [3, 6].

Despite recognition of these barriers, transformations to improve type-2 diabetes care and outcomes among First Nations people have been slow. Systematic proactive organization of diabetes care for First Nations people with diabetes living on reserves is lacking in Canada [2]. Current practice guidelines and Aboriginal Diabetes Initiative’s in Canada call for innovative, culturally relevant approaches that rely on community-specific programs for people with type-2 diabetes developed and delivered in partnership with target communities [2, 8].

Information systems that allow for a comprehensive-based approach to care, such as electronic health records (EHRs) linked with diabetes registries, positively impact quality of diabetes care and reduce costs [11–13]. Furthermore, incorporation of professional support to fully leverage the infrastructure, through a care coordinator, has been shown to improve intermediate measures of cardiometabolic care (e.g., A1c, blood pressure, cholesterol) and many other processes of care (e.g., foot and eye care, chronic care coordination) compared to usual care, however delivered [9, 11–16]. Currently, few First Nations communities have the infrastructure, expertise, or resources to employ the 5R’s as part of their CCM based quality improvement strategies, particularly the foundation upon which all these efforts are built: the ability to systematically identify and track diabetes patients and share information seamlessly (i.e., **R**egister, **R**elay, **R**ecall). Thus, it is unknown whether these benefits apply to the Canadian context and specifically to remote, isolated and under-resourced First Nations communities. This is context for which the “Reorganizing the Approach to Diabetes through the Application of Registries” (RADAR) project was designed.

### Objectives

Our purpose is to describe the RADAR project. Our primary objective is to implement and assess the effectiveness of an integrated population based electronic health record (EHR)/registry system specifically designed for First Nations communities (*Community Assessment Response and Empowerment [CARE] platform),* coupled with dedicated support from a centralized care coordinator (CC) to systematically organize proactive diabetes care in the communities to improve diabetes-related outcomes. Thus, RADAR is the combination of the CARE platform and the CC support. We hypothesize that RADAR will result in an overall improvement in the quality of clinical care of First Nations people living with type-2 diabetes on reserve, as measured by evidence-based outcomes (A1c, BP, cholesterol) and quality of care indicators (e.g. foot and eye examinations). We believe that RADAR will help promote timely management of people with type-2 diabetes and increase diabetes knowledge and management practices among local healthcare workers [15, 17]. In addition, managers can use the EHR/registry to track diabetes trends, determine the effectiveness of diabetes programs, and assist in resource planning.

## Methods

### Model overview

The RADAR model incorporates two distinct elements: 1) an EHR, integrated with a population-based diabetes registry and analytics platform – *CARE platform*; and, 2) a centralized care coordinator. The CARE platform was designed by OKAKI Health Intelligence Inc. to meet the specific requirements of nursing-driven health programs currently being delivered on reserve in Canadian First Nations communities. Major limitations of existing EHRs, which are largely designed for physician offices, are: misalignment with programs, reporting and professional practice needs of service providers on reserve; no population-based registry that supports sharing between facilities and population (as well as individual) measurement and chronic disease management; and cost [18, 19].

The first element of RADAR, the CARE platform, specifically integrates a patient registry and electronic clinical chart for patients across three traditionally distinct and segregated programs responsible for care on reserves (Home and Community Care, Aboriginal Diabetes Initiative, and Community Health); thereby, improving coordination of care between service providers on reserve. The CARE platform includes a diabetes registry, enabling the systematic, standardized capture of patient data on key diabetes outcomes and quality of care indicators (e.g., A1c, BP, cholesterol, foot and eye exams); and incorporates performance-based measurement and reporting on clinical care standards and targets at both the population and individual level. The system also provides real-time recognition and recall of patients needing follow-up care. Moreover, the CARE platform uses a simple interface and can be accessed through a web-based portal allowing for remote monitoring and evaluation of care and reporting.

In our ongoing discussions with community leaders, most diabetes care tends to be reactive to clients seeking care rather than proactively in accordance with clinical practice guidelines. Many clients do not receive ongoing diabetes care and do not have a regular general practitioner who coordinates care. Thus, we have combined our CARE platform with a key facilitator – a centralized care coordinator (CC). The CC is ideally a First Nations registered nurse and certified diabetes educator with significant experience with care on reserve. The CC will work remotely with the local healthcare workers on reserve to improve quality of care for people with diabetes, facilitated through shared use of the CARE platform and web conferencing technologies. The CC supports systematic review of patients and case conferencing activities to support and prioritize care, problem-solves system or patient-level care issues, and provides education to local healthcare workers. In addition, the CC acts as a bridge between the First Nations communities and other service providers to help coordinate patient care on and off reserve. Together, both RADAR elements address the 5Rs in organizing diabetes care, that is, recognize, register, relay, recall, and resource, in the First Nations context. In addition, managers and researchers can use the analysis of population-level data for epidemiological assessments, quality-improvement, and decision-making.

### Setting

RADAR involves collaboration with several First Nations communities in Alberta, representing separate treaty areas across the province (continuous expansion of the RADAR model to additional First Nations communities is ongoing). We estimate that approximately 1000 people with type-2 diabetes currently reside in the pilot communities. All communities involved are several hours of travel from a major urban center, where the majority of diabetes specialist care services are located. Some communities have local family physician clinics, located just off reserve that also provide care for patients with diabetes; other communities have visiting family physicians on one or more days a week; while other communities have no local physician services, although services are available in neighboring rural communities. As a result, local, on-reserve, healthcare workers are the primary service providers delivering diabetes care and general health services for these populations. Prior to implementing RADAR, all communities were familiar with the CARE platform for the management of patient care within their respective communities.

### Outcomes

The primary outcome will be a 10% improvement in measures over the baseline control periods in any one of the following: A1c, BP, cholesterol. This is consistent with our previous Canadian work that considers a 10% improvement in any of these measures to be the minimally clinically important difference [20]. For patients who are already at ‘target’ for any or all three measures at baseline, we will consider persistence at target (i.e., maintain values within 10% of baseline) as also achieving the primary outcome. Secondary endpoints will address other areas of diabetes care and process indicators compared to baseline including: the proportion of clinical measures and tasks completed in accordance with Canadian Diabetes Association Clinical Practice guidelines (e.g., A1c, BP, cholesterol, foot and eye examination, receipt of vaccinations, smoking cessation counseling); the number of patients registered in CARE; and the proportion of patients linked to a health services provider. In addition, we are collecting detailed information to conduct a comprehensive economic evaluation, assessing the cost-effectiveness of RADAR specific to these communities. Our approach to the economic analysis will be analogous to methods previously published by our group [21, 22].

We will also conduct a qualitative assessment as part of our comprehensive evaluation to supplement the primary and secondary quantitative assessments. The purpose of the qualitative component is to provide contextual information, such as the quality/usability of the CARE platform and the impact/satisfaction of local healthcare workers with the CC model.

### Eligibility and recruitment

Participation in RADAR is dictated by the Health Director and leadership within each First Nations community health facility. After agreeing to participate, local healthcare workers (e.g., Health Center, Healing Center) will actively register First Nations patients with diabetes into the CARE platform. All patients in the community are eligible to be included in the CARE platform; however, patients must meet the following criteria to be evaluated within RADAR:

- *Inclusion criteria*: Patients diagnosed with type-2 diabetes; recently (within last 1-2 years) received care from the First Nations health facility; provided verbal consent for First Nations health care workers to manage their diabetes.
- *Exclusion criteria*: Patients with type-1 diabetes; patients <18 years of age; those who are subsequently discovered not to have type-2 diabetes; those refusing care within the First Nations health facility; patients with type-2 diabetes identified after the conclusion of the baseline phase of the project.

### Implementation and evaluation

**Fig. 1:**
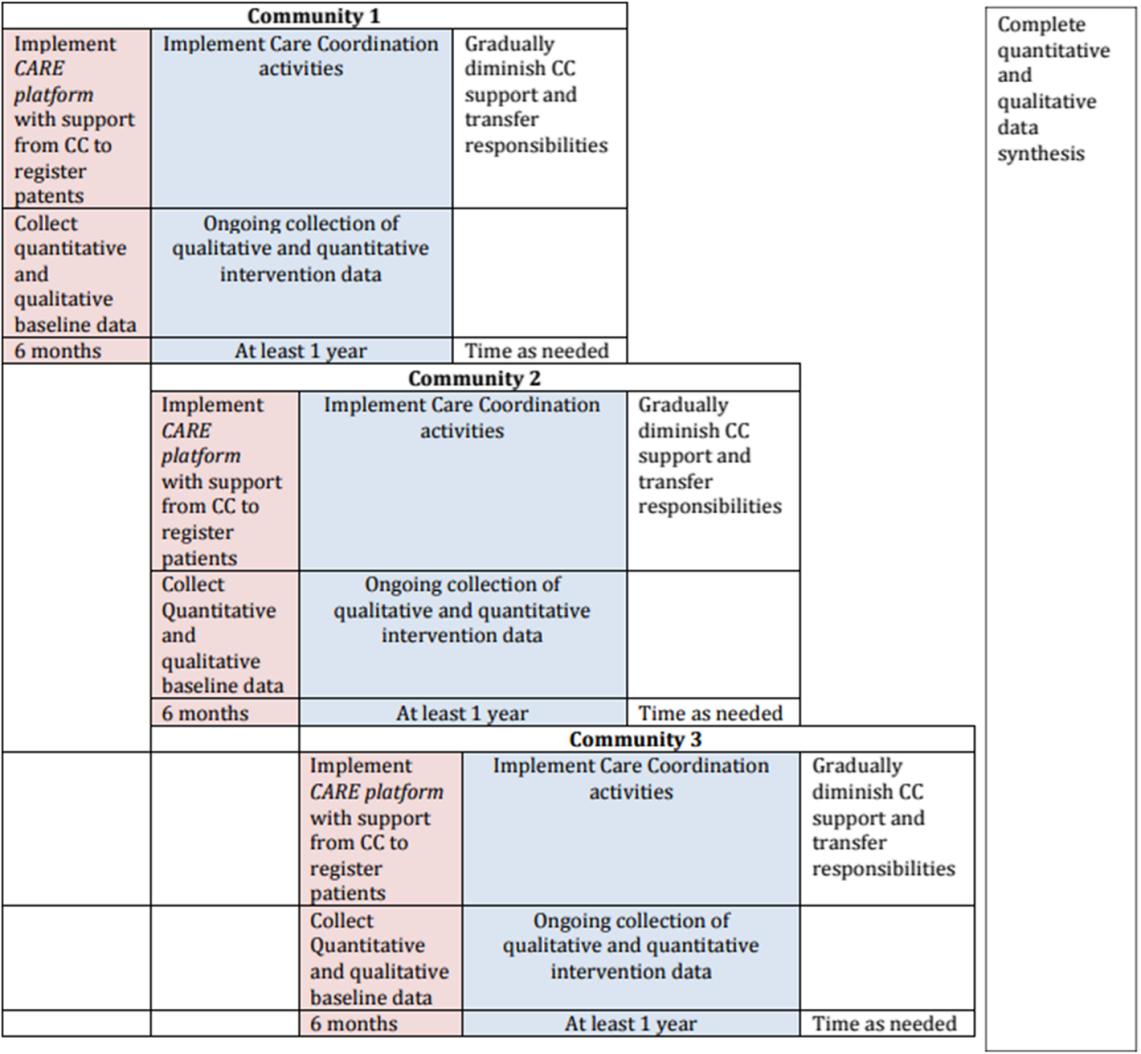
RADAR Stepped-Wedge Design

The implementation and evaluation of RADAR is segregated into two distinct parts. OKAKI Health Intelligence Inc., who has a long history of working with First Nations communities in the province, will implement RADAR, including the CARE platform and care coordination. Researchers from the University of Alberta will independently evaluate the effectiveness of RADAR. By separating the implementation from the evaluation, we believe a more robust evaluation will be achieved. To ensure a robust evaluation, a modified stepped wedge controlled implementation trial design will be employed. This design is a highly valid cross-over design, whereby each community serves as its own control before ‘switching’ to the intervention at different time points [23, 24]. In essence, RADAR will be implemented sequentially to the communities in consecutive 4–6 month periods, based on the communities ‘readiness’. By the end of the evaluation period, RADAR will have been implemented in all communities (Fig. 1).

### Implementation design

#### Baseline ‘control’ phase

Once a community has been allocated to begin RADAR, the community will enter into an initial 6-month prospective baseline phase. During the baseline phase, the CC will work with the local healthcare workers to recognize all known patients with diabetes through existing medical records and histories, ad-hoc registries, etc. All newly identified patients with type-2 diabetes discovered during the baseline phase will also be included. Once identified, all patients will be registered into the CARE platform, including their demographic and clinical data (e.g., A1c, blood pressure, lipids, eye exams, foot care), where available. Patient information to populate the baseline data will be obtained from all potential sources (e.g., medical records, patient interviews, Alberta’s provincial EHR for laboratory results (Netcare)). All available data up to 18 months prior to the end of the baseline period will be used to populate the CARE platform, as patient encounters with the health system may not be consistent. Patients identified with type-2 diabetes following the conclusion of the baseline phase will be included in the CARE platform and process of care coordination but will not be included in analyses, as no baseline control phase will be available for these patients. Importantly, although the CC will work with the local healthcare workers to recognize and register patients with type-2 diabetes, the CC will not make care management recommendations during the baseline phase.

### Intervention phase

After completion of the *Baseline Phase*, each community will transition into the *intervention phase* for a minimum of 1 year. The intervention phase will conclude on December 31, 2020, for all communities and length of follow-up is dependent on timing of initiation of RADAR within the community. During the intervention phase, the CC will help the local healthcare workers to proactively manage patients with type-2 diabetes, including monitoring and recall of patients, relaying clinical information and coordinating care, facilitated through shared use of the CARE platform and web conferencing technologies. In addition, the CC will provide support on the use of the CARE platform and training and education for the local healthcare workers on ‘best practices’ for the management of type-2 diabetes. It is expected that the local healthcare workers’ reliance on the CC will diminish over time, as they become more confident and competent using the CARE platform and in their diabetes knowledge and ability to manage patients, leading to sustainability.

### Evaluation design

We will conduct a comprehensive evaluation of RADAR, using the RE-AIM framework [25]. We have used this framework to evaluate other diabetes-related, quality-improvement interventions [26]. RE-AIM consists of five dimensions: **R**each into the target population; **E**ffectiveness of the intervention; **A**doption by targeted end-users; **I**mplementation, including consistency and cost of delivery; and **M**aintenance of intervention effects over time [25] Table 1.

Thus, our comprehensive evaluation will include both quantitative and qualitative components. We will use the quantitative data for the clinical outcomes mentioned above as part of our overall assessment of the dimension of effectiveness. The primary source of qualitative data includes interviews during the baseline, intervention and post intervention phases. We will use purposeful sampling to identify key informants with expert knowledge of the implementation of RADAR in each community (e.g. the CC, local healthcare workers, and health directors). All interviews will take place at community health facilities or via videoconferencing, using semi-structured interview guides. The purpose of the interviews is to document the organization of existing type-2 diabetes care within each community, implementation facilitators, barriers, and lessons learned, perceived appropriateness of RADAR for the communities, and the overall usability and acceptance of the technology (i.e., CARE platform). In addition, the software vender will also capture changes made to the software over time as requested by the communities. The interviews will be digitally recorded for subsequent analysis, transcribed verbatim by an independent transcriptionist, and verified for accuracy. All qualitative data sources (e.g. validated baseline assessments and interview transcripts) will be compiled and managed using Atlas.ti 7 software.

### Data analysis

For quantitative data, all relative changes will be analyzed using the last known recorded value from the CARE platform from baseline to the end of the intervention phase. We will employ an intention-to-treat analysis, whereby all patients who are enrolled and have baseline data will be included in our primary analysis. The primary endpoint (i.e., 10% improvement in any of A1c, BP, cholesterol values from baseline or those maintaining values within 10% of baseline if at any or all three targets at baseline) will be evaluated using a conventional intention to treat analysis with logistic regression. However, if the observed intracluster correlation coefficient (ICC) is non-ignorable, we will use generalized estimating equations (GEE) that accounts for the clustered and hierarchical nature of our data [24]. Several a-priori subgroup analyses are planned for the primary endpoint. First, we will restrict analyses to patients not at target for any of the 3 primary outcome measures. Second, we will restrict analyses to those patients who were at target for each primary outcome measure (A1c, BP, cholesterol) to fully examine persistence at target over time. Additional subgroups of interest include analyses stratified by sex, and analyses stratified by age (<65 vs > =65 years). Secondary endpoints include: the proportion of clinical measures completed in accordance with guidelines (e.g., A1c, BP, cholesterol, foot and eye examination, receipt of vaccinations, smoking cessation counseling); the number of patients registered in CARE; and the proportion of patients linked to a health services provider.

We anticipate not having complete quantitative data on all endpoints of interest, given the real-world implementation of the project. Several approaches will be used for missing clinical data. First, in alignment with the intention-to-treat approach, we will use the baseline observation carried forward technique for our primary outcome. The stability of the results will be additionally evaluated using the multiple imputation method as a sensitivity analysis.

For qualitative data analysis, we will use an integrated approach. First, we will use the RE-AIM framework as the initial coding structure. Second, we will use an inductive approach [27] to identify emerging codes and concepts within each dimension using conventional approaches [28–30]. We will employ several verification strategies to ensure the reliability and validity of the qualitative results including methodological coherence, appropriate sampling, collecting and analyzing data concurrently [31], and debriefing [30].

### Power and sample size

In prior studies, about 40% of diabetes patients will have at least a 10% improvement in A1c, BP, and cholesterol over time when studied under ‘usual care’[32]. To detect at least an additional 10% improvement in the proportion of patients achieving our primary endpoint under the RADAR model (i.e., move the population from 40% achieving endpoint to at least 50% achieving endpoint), at an alpha =0.05, an ICC of 0.01, we require 1153 total patients to detect a clinically important difference with 80% power. Assuming a 10% loss to follow-up, we require approximately 1250 total patients among our communities.

## Discussion

It is well known that diabetes care is suboptimal in First Nations populations [4–6]. Our own research [20, 32], coupled with other large-scale research projects across the country, has clearly demonstrated the need for improved care in this high-risk population [5, 6]. We will evaluate the effectiveness of RADAR in improving the quality of care for First Nations people with type-2 diabetes living on reserve. The aim of RADAR is to improve processes of care, and ultimately clinical outcomes in patients, and to build local capacity and expertise in the management of diabetes. The current Canadian Diabetes Association Clinical Practice Guidelines strongly advocate for multidisciplinary teams to improve outcomes in people with diabetes [8], yet these multidisciplinary teams are often lacking in First Nations communities [5]. We believe RADAR, including the CARE platform and CC model, can facilitate a culturally relevant community-based multidisciplinary team approach to diabetes care.

There are several strengths related to our implementation and evaluation of RADAR. First, the RADAR model is well positioned to succeed in the communities based on prior work by our team. OKAKI Health Intelligence Inc. has been successfully working in First Nations communities in the province for over 7 years. This partnership reinforces that RADAR is community-specific, culturally relevant and promotes its sustainability. Moreover, the communities have already adopted the CARE platform for other aspects of patient care (e.g., home care). Therefore, the extension of the CARE platform to chronic disease management is highly feasible.

Second, our use of the RE-AIM framework will provide insight into the broader impact of RADAR beyond clinical effectiveness and identify both facilitators and barriers to implementing RADAR within each community. These results will help inform scale and spread of RADAR to other suitable communities and populations.

Third, we will employ a valid stepped wedge study design. Often quality-improvement projects show benefit when evaluated, but are conducted without a robust comparison group, making assessment of the true absolute improvements in care difficult to decipher. By including control periods over time, we will be able to elucidate any improvements directly attributed to the RADAR model.

Regardless, there are design features of RADAR that warrant further discussion. First, we have used process (i.e., intermediate or surrogate) outcomes as measures of quality, rather than harder clinical endpoints (e.g. diabetic complications like amputation, heart attack or stroke). We chose these process endpoints, as we do not expect to observe changes in hard clinical outcomes over the short timeline of this project. Nevertheless, reductions in these outcomes (e.g. A1c, BP, cholesterol, etc.) are well established in many large scale RCTs as conferring substantial benefits on either macro- or microvascular outcomes and are recommended in the Canadian Diabetes Association Clinical Practice Guidelines [8, 32]. Moreover, our objective is to determine if RADAR can influence these clinical measures, not reaffirm if these changes are important on hard clinical endpoints. If the intervention improves these outcomes, hard clinical endpoints would be expected to also change (e.g. heart attacks and stroke) over time.

Second, our economic models cannot be fully populated by our results. This is true of most models and we will need to make assumptions based on literature and expert opinion. However, our proposed economic approach is peer-reviewed and published and we have used it in previous analogous analyses [21, 22]. We will also undertake many sensitivity analyses to demonstrate the results are robust and valid.

Last, we have designed a pragmatic and feasible controlled project rather than a randomized controlled efficacy trial. Our project is a community-based intervention aimed at the local healthcare workers as opposed to patients *per se*. Individual patients could not be randomized to care with and without RADAR because of the threat of “contamination” (i.e., patients within a community health facility would likely be exposed to some aspects of the intervention regardless of study arm). Similarly, randomization could not occur at the level of the local healthcare workers for similar reasons (e.g., increased knowledge on the appropriate care of patients with diabetes would be expected to diffuse throughout the entire care team in a health facility). Moreover, acceptance of a community to a traditional randomized control trial would be expected to be low, as few communities would be accepting of being the control community for extended durations. The stepped wedge design will be morally and politically more acceptable to the communities, and because of logistical, practical, and financial constraints, it can be better implemented in stages. Furthermore, the proposed design incorporates a number of methods other than random allocation to protect internal validity (e.g., controlled before after with sequential roll-out to reduce time-related biases), and the design is considered valid and included in the Cochrane EPOC Systematic Reviews. The results from our project may have greater applicability than the results of a randomized trial in a highly selected and potentially biased sample.

## Conclusion

RADAR combines innovative technology with personalized support to deliver organized diabetes care in remote First Nations communities in Alberta. The project is novel as it involves public, academia and private sector partnerships to improve care for First Nations people with type-2 diabetes. By design, our integrated EHR/registry connects all personnel/facilities and enables real time analysis at community and system levels. Moreover, care coordination typically refers to a single person in the community to oversee care, which would be difficult to replicate or build capacity for all First Nations communities. In our innovative approach, the local healthcare workers and centralized CC will work together to support patient and community health needs, using technology to support communication. Thus, each healthcare worker focuses on his or her own strengths, connections and capacity to improve care. Moving forward, hopefully RADAR, in combination with other similar initiatives, may bridge the gap between First Nations health and the general population, and reduce diabetes-related morbidity and premature mortality.

## Abbreviations

BP: Blood pressure
CC: Care coordinator
CCM: Chronic care model
EHR: Electronic health record
RADAR: Reorganizing the approach to diabetes through the application of registries
RE-AIM: Reach; effectiveness; adoption; implementation; maintenance

## References

[1] Naqshbandi M, Harris SB, Esler JG, Antwi-Nsiah F (2008). Global complication rates of type 2 diabetes in Indigenous peoples: A comprehensive review. Diabetes Res Clin Pract.

[2] Aboriginal Diabetes Initiative (Canada). Accountability and Evaluation Working Group., Canada. Health Canada. Aboriginal Diabetes Initiative : evaluation framework. Ottawa: Health Canada; 2002. p. 32.

[3] Oster RT, Toth EL, King M, Crowshoe L (2011). Diabetes and The Status Aboriginal Population in Alberta. Alberta Diabetes Atlas 2011.

[4] Bailie RS, Si D, Robinson GW, Togni SJ, D'Abbs PH (2004). A multifaceted health-service intervention in remote Aboriginal communities: 3-year follow-up of the impact on diabetes care. Med J Aust.

[5] Oster RT, Virani S, Strong D, Shade S, Toth EL (2009). Diabetes care and health status of First Nations individuals with type 2 diabetes in Alberta. Can Fam Physician.

[6] Harris SB, Naqshbandi M, Bhattacharyya O, Hanley AJ, Esler JG, Zinman B (2011). Major gaps in diabetes clinical care among Canada's First Nations: results of the CIRCLE study. Diabetes Res Clin Pract.

[7] Gracey M, King M (2009). Indigenous health part 1: determinants and disease patterns. Lancet.

[8] Canadian Diabetes Association Clinical Practice Guidelines Expert Committee (2013). Canadian Diabetes Association 2013 Clinical Practice Guidelines for the Prevention and Management of Diabetes in Canada. Can J Diabetes.

[9] Saxena S, Misra T, Car J, Netuveli G, Smith R, Majeed A (2007). Systematic review of primary healthcare interventions to improve diabetes outcomes in minority ethnic groups. J Ambul Care Manage.

[10] Adelson N (2005). The embodiment of inequity: health disparities in aboriginal Canada. Can J Public Health.

[11] Shojania KG, Ranji SR, McDonald KM, Grimshaw JM, Sundaram V, Rushakoff RJ (2006). Effects of quality improvement strategies for type 2 diabetes on glycemic control: a meta-regression analysis. Jama.

[12] Tricco AC, Ivers NM, Grimshaw JM, Moher D, Turner L, Galipeau J (2012). Effectiveness of quality improvement strategies on the management of diabetes: a systematic review and meta-analysis. Lancet.

[13] Grant RW, Hamrick HE, Sullivan CM, Dubey AK, Chueh HC, Cagliero E (2003). Impact of population management with direct physician feedback on care of patients with type 2 diabetes. Diabetes Care.

[14] Borgermans L, Goderis G, Van Den Broeke C, Verbeke G, Carbonez A, Ivanova A (2009). Interdisciplinary diabetes care teams operating on the interface between primary and specialty care are associated with improved outcomes of care: findings from the Leuven Diabetes Project. BMC Health Serv Res.

[15] van Bruggen R, Gorter K, Stolk R, Klungel O, Rutten G (2009). Clinical inertia in general practice: widespread and related to the outcome of diabetes care. Fam Pract.

[16] Davidson MB, Blanco-Castellanos M, Duran P (2010). Integrating nurse-directed diabetes management into a primary care setting. Am J Manag Care.

[17] Sperl-Hillen B, Palattao AK (2010). utpatient EHR-based diabetes clinical decision support that works: lessons learned from implementing diabetes wizard. Diabetes Spectrum.

[18] Hoffman S, Podgurski A (2013). Big bad data: law, public health, and biomedical databases. J Law Med Ethics.

[19] Kukafka R, Ancker JS, Chan C, Chelico J, Khan S, Mortoti S (2007). Redesigning electronic health record systems to support public health. J Biomed Inform.

[20] Majumdar SR, Guirguis LM, Toth EL, Lewanczuk RZ, Lee TK, Johnson JA (2003). Controlled trial of a multifaceted intervention for improving quality of care for rural patients with type 2 diabetes. Diabetes Care.

[21] Johnson JA, Lier DA, Soprovich A, Al Sayah F, Qiu W, Majumdar SR (2016). Cost-Effectiveness Evaluation of Collaborative Care for Diabetes and Depression in Primary Care American journal of preventive medicine.

[22] Johnson ST, Lier DA, Soprovich A, Mundt C, Johnson JA (2015). How much will we pay to increase steps per day? Examining the cost-effectiveness of a pedometer-based lifestyle program in primary care. Prev Med Reports.

[23] Handley MA, Schillinger D, Shiboski S (2011). Quasi-experimental designs in practice-based research settings: design and implementation considerations. J Am Board Fam Med.

[24] Hussey MA, Hughes JP (2007). Design and analysis of stepped wedge cluster randomized trials. Contemp Clin Trials.

[25] Glasgow RE, Vogt TM, Boles SM (1999). Evaluating the public health impact of health promotion interventions: the RE-AIM framework. Am J Public Health.

[26] Wozniak L, Rees S, Soprovich A, Al Sayah F, Johnson ST, Majumdar SR, et al. Applying the RE-AIM framework to the Alberta’s Caring for Diabetes Project: a protocol for a comprehensive evaluation of primary care quality improvement interventions. BMJ open. 2012;2(5). Epub 2012/10/30. doi:10.1136/bmjopen-2012-002099. PubMed PMID: 23103609; PubMed Central PMCID: PMC3488740. 10.1136/bmjopen-2012-002099 PMC3488740 23103609

[27] Thomas DR (2006). A general inductive approach for analyzing qualitative evaluation data. Am J Eval.

[28] Hsieh HF, Shannon SE (2005). Three approaches to qualitative content analysis. Qual Health Res.

[29] Mayan MJ (2009). Essentials of qualitative inquiry.

[30] Morse JM, Field PA (1995). Qualitative Research Methods for Health Professionals.

[31] Morse JM, Barret M, Mayan M, Olson K, Spier J (2002). Verification strategies for establishing reliability and validity in qualitative research. Int J Qual Methods.

[32] Johnson JA, Eurich DT, Toth EL, Lewanczuk RZ, Lee TK, Majumdar SR (2005). Generalizability and persistence of a multifaceted intervention for improving quality of care for rural patients with type 2 diabetes. Diabetes Care.

[33] Canadian Institutes of Health Research., Interagency Advisory Panel on Research Ethics (Canada), Interagency Secretariat on Research Ethics (Canada), Natural Sciences and Engineering Research Council Canada., Social Sciences and Humanities Research Council of Canada. Tri-council policy statement ethical conduct for research involving humans. [Ottawa, Ont.]: [Interagency Secretariat on Research Ethics]; 2010. Available from: http://www.deslibris.ca/ID/227500

